# The Clinical Impact of Imaging Surveillance and Clinic Visit Frequency after Acute Aortic Dissection

**DOI:** 10.1055/s-0039-1692187

**Published:** 2019-10-15

**Authors:** Ashish Chaddha, Kim A. Eagle, Himanshu J. Patel, G. Michael Deeb, Bo Yang, Kevin M. Harris, Alan C. Braverman, Stuart Hutchison, Arturo Evangelista, Rossella Fattori, James B. Froehlich, Christoph A. Nienaber, Eric M. Isselbacher, Dan G. Montgomery, Eva Kline-Rogers, Elise Woznicki, Troy M. LaBounty

**Affiliations:** 1 Department of Medicine, University of Michigan, Ann Arbor, Michigan; 2 Department of Surgery, University of Michigan, Ann Arbor, Michigan; 3 Department of Medicine, Minneapolis Heart Institute, Minneapolis, Minnesota; 4 Department of Medicine, Washington University, St. Louis, Missouri; 5 Department Medicine, University of Calgary, Calgary, Canada; 6 Department of Medicine, Hospital General Universitari Vall d'Hebron, Barcelona, Spain; 7 Department of Medicine, University Hospital S. Orsola, Bologna, Italy; 8 Department of Medicine, University of Rostock, Rostock, Germany; 9 Massachusetts General Hospital, Harvard Medical School, Boston, Massachusetts

**Keywords:** aortic diseases, aorta, diagnostic imaging

## Abstract

**Background** Guidelines recommend frequent follow-up after acute aortic dissection (AAD), but optimal rates of follow-up are not clear.

**Methods** We examined rates of imaging and clinic visits in 267 individuals surviving AAD during recommended intervals (≤1, > 1–3, > 3–6, > 6–12 months, then annually), frequency of adverse imaging findings, and the relationship between follow-up and mortality.

**Results** Type A and B AAD were noted in 46 and 54% of patients, respectively. Mean follow-up was 54.7 ± 13.3 months, with 52 deaths. Adverse imaging findings peaked at 6 to 12 months (5.6%), but rarely resulted in an intervention (3.4% peak at 6–12 months). Compared with those with less frequent imaging, patients with imaging for 33 to 66% of intervals ( *p* = 0.22) or ≥66% of intervals ( *p* = 0.77) had similar adjusted survival. In comparison to patients with fewer clinic visits, those with visits in 33 to 66% of intervals experienced lower adjusted mortality (hazards ratio: 0.47, 95% confidence interval: 0.23–0.97, *p* = 0.04), with no difference seen in those with ≥66% (vs. < 33%) interval visits ( *p* = 0.47). Imaging at 6 to 12 months (vs. none) was associated with decreased adjusted mortality (hazards ratio: 0.50, 95% confidence interval: 0.27–0.91, *p* = 0.02), while imaging during other intervals, or clinic visits during any specific intervals, was not associated with a difference in mortality ( *p* > 0.05 for each).

**Conclusions** Adverse imaging findings following AAD are common, but rarely require prompt intervention. Patients with the lowest and highest rates of clinic visits experienced increased mortality. While the overall rate of surveillance imaging did not correlate with mortality, adverse imaging findings and related interventions peaked at 6 to 12 months after AAD, and imaging during this time was associated with improved survival.

## Introduction

Patients surviving acute aortic dissection (AAD) have a significant risk of morbidity and mortality following discharge. 1 2 Long-term management of these patients includes follow-up clinic visits for assessment of symptoms, blood pressure control, smoking cessation, and lifestyle modifications, as well as surveillance imaging to identify anatomic changes in the aorta. Both American and European consensus guidelines recommend surveillance imaging of the postdissection aorta at intervals of 1, 3, 6, and 12 months, and then annually in patients with stable findings. European guidelines also endorse regular outpatient visits at these same intervals by the physicians specialized in managing patients with aortic dissection. 3 4 These guidelines are largely based on expert opinion, and there is a lack of data establishing the need and clinical utility of such frequent follow-up. We examined patterns of clinic visit and imaging follow-up after discharge for AAD, and evaluated the prevalence and clinical impact of adverse imaging findings. We hypothesized that low rates of follow-up after discharge for AAD may be associated with increased mortality.

## Materials and Methods

This retrospective study examined consecutive adult individuals presenting with AAD at a major enrolling site in the International Registry of Acute Aortic Dissection. The details of this registry have previously been described in detail. 5 6 7 All patients were enrolled between January 1, 1996 and November 1, 2011. Type A AAD was defined as any nontraumatic dissection involving the ascending aorta and presenting within 14 days of symptom onset. Type B AAD was defined as any nontraumatic dissection involving the descending aorta and presenting within 14 days of symptom onset. Patients were identified either prospectively at presentation or retrospectively via discharge diagnoses, imaging, and surgical databases. Diagnosis was based on imaging or surgical visualization. A total of 92 of 267 patients (34%) experienced AAD prior the publication of guidelines endorsing imaging and clinic visit follow-ups at intervals of 1, 3, 6, and 12 months, then annually. This study was approved by the Institutional Review Board with a waiver of informed consent.

For this study, inclusion criteria included adult patients presenting with AAD who survived to discharge ( *n* = 425). We excluded individuals who lived > 500 miles from the enrolling site ( *n* = 23) as they would be less likely to have follow-up at the index hospital. All clinical records were carefully reviewed for any indication that patients had partial imaging or specialized clinic visits at other sites, and all patients with partial follow-up at other sites were also excluded ( *n* = 11). Finally, we excluded those without at least one imaging test and one follow-up clinic visit after discharge ( *n* = 124), resulting in a total of 267 patients for the study.

A standardized baseline form was used to record clinical variables, which included information on patient demographics, history, clinical presentations, aortic imaging findings, management, and patient outcomes. All-cause mortality was assessed using the Social Security Death Index and using the electronic medical record based on its query of state and federal death records. Supplemental review of the electronic medical record was used to determine patterns of imaging surveillance and clinic visit follow-up, and records were reviewed to determine blood pressure, rates of tobacco cessation, and adverse imaging findings. Clinic visits were limited to specialized follow-up with a cardiothoracic surgeon, vascular surgeon, cardiovascular physician, or other physician specialized in aortic dissection as recommended per guidelines. 4 Imaging follow-up included transesophageal echocardiography (TEE), computed tomographic (CT) angiography, and/or magnetic resonance angiography (MRA). Adverse imaging findings were defined as any adverse imaging finding on the clinical reports, and included new dissection, false lumen enlargement, new aneurysm or increased aortic dilatation, new endograft leak, new intramural hematoma, new pseudoaneurysm, new penetrating ulcer expansion, new dilation of branch artery, new partial thrombosis of the false lumen, or other aortic or related change on imaging. In all cases, images were directly compared with the prior study as well as earlier studies to assess for any interval change using multiplanar reformats as appropriate, and all patients had index studies available for comparison. Increased false lumen enlargement and aortic dilatation were based on any increase ≥1 mm in size compared with the prior studies on direct side-by-side comparison; differences in measurements that were considered within measurement error by the radiologist were not counted as adverse imaging findings (in all cases, these did not exceed 1 mm difference from the prior study). Imaging findings that prompted interventional procedures were defined as an adverse imaging finding that was documented in the medical record to prompt open surgery of the aorta or endovascular aorta repair.

Comparisons in mean blood pressure were performed between individuals with < 33%, 33 to 66%, and > 66% clinic visit follow-up, using the mean of all available blood pressure results following discharge and at expected intervals listed below. Rates of tobacco use were compared between index admission and the date of last follow-up.

All imaging studies were interpreted by nonblinded, experienced, and specialized clinical readers. CT and MRA studies were interpreted by board-certified fellowship-trained cardiothoracic radiologists, and TEE studies were interpreted by cardiologists with dedicated fellowship training in echocardiography and level III certification for echocardiography. All readers had at least several years of experience interpreting studies with aortic pathology including aortic dissection.

We examined rates of adherence to guidelines for imaging and clinic visit follow-up at defined intervals of ≤1, > 1 to 3, > 3 to 6, > 6 to 12, > 12 to 24, > 24 to 36, > 36 to 48, and > 48 to 60 months. Analyses for imaging surveillance and clinic visit follow-up were performed separately, with patients censored after their last imaging test or clinic visit follow-up for each analysis, as we could not exclude the possibility that patients may have had imaging and clinic visits at other sites after that time.

Patients were divided into three groups based on percentage of imaging or clinic visits observed within their available follow-up data (< 33, 33–66, or > 66%), with all analyses done separately for imaging and clinic visits, respectively. Comparisons between these three groups were performed using chi-square tests and *t* -tests for categorical and continuous variables, respectively.

Kaplan–Meier analysis and log-rank tests were used to determine whether different rates of imaging surveillance or clinic visits were associated with differences in survival after discharge. After univariate analysis to identify variables related to mortality, candidate variables were selected with *p* -values less than 0.20 to introduce to a multivariable analysis. Cox proportional hazards analysis was performed using a backward stepwise method to determine the independent relationship between follow-up groups and mortality. Considered variables included age > 65 years, Type B versus Type A aortic dissection, gender, comorbidities such as hypertension, atherosclerosis, aortic aneurysm, bicuspid aortic valve, iatrogenic dissection, prior coronary angiography, coronary artery disease, heart failure, chronic renal disease, aortic insufficiency, ischemic spinal cord injury, cerebrovascular accident, electrocardiographic findings such as low voltage and prior Q waves, chest X-ray findings such as aortic calcification and pleural effusion, and presenting symptoms such as anterior chest pain, leg pain, and syncope. A *p* -value of < 0.05 was considered statistically significant. IBM SPSS Statistics Version 22.0 (IBM Corp., Armonk, NY) was used for analysis.

## Results

The study group included 267 individuals. Patient demographics, past medical history, dissection type, and dissection management are provided in Tables 1 and 2 , and are stratified by < 33%, 33 to 66%, and > 66% follow-up. Surgery or endovascular repair were performed in 80 (99/123) and 40% (57/144) of individuals with Type A and Type B AAD, respectively, prior to discharge. Inpatient events, including stroke or transient ischemic attack, myocardial infarction or ischemia, and acute renal failure, were not different between groups ( *p* > 0.10 for each).

**Table 1:** Patient characteristics stratified by proportion of interval surveillance imaging performed after acute aortic dissection

Variables	Overall *n* = 267	< 33%, *n* = 80 (30%)	33–66%, *n* = 86 (32%)	> 66%, *n* = 101 (38%)	*p* -Value
*Demographics:*					
Age (y)	59.9 ± 14.3	62.2 ± 15.1	58.0 ± 14.0	59.8 ± 13.7	0.16
Female gender	91 (34%)	28 (35%)	33 (38%)	30 (30%)	0.45
Caucasian (vs. other)	202 (77%)	54 (68%)	64 (76%)	84 (86%)	**0.02**
Distance to home (miles)	70.9 ± 61.4	74.5 ± 76.0	72.0 ± 49.4	67.3 ± 58.0	0.73
*Past medical history:*					
Marfan syndrome	17 (7%)	2 (3%)	4 (5%)	11 (11%)	0.07
Hypertension	202 (76%)	63 (80%)	69 (80%)	70 (70%)	0.18
Aortic aneurysm	37 (14%)	8 (10%)	12 (14%)	17 (17%)	0.45
Coronary artery disease	81 (31%)	23 (30%)	28 (33%)	30 (30%)	0.88
Bicuspid aortic valve	10 (4%)	2 (3%)	4 (5%)	4 (4%)	0.79
Prior aortic dissection	21 (8%)	3 (4%)	6 (7%)	12 (12%)	0.14
Mitral valve disease	9 (6%)	3 (7%)	3 (8%)	3 (5%)	0.83
Diabetes	19 (7%)	9 (12%)	4 (5%)	6 (6%)	0.22
Current smoking	76 (36%)	24 (39%)	25 (40%)	27 (31%)	0.45
Cocaine abuse	13 (5%)	4 (5%)	6 (7%)	3 (3%)	0.46
Renal insufficiency	7 (5%)	4 (9%)	2 (5%)	1 (2%)	0.23
PCI	11 (5%)	4 (5%)	4 (5%)	3 (3%)	0.93
Aortic valve disease	29 (11%)	7 (9%)	11 (13%)	11 (11%)	0.74
Emphysema	24 (17%)	7 (16%)	8 (21%)	9 (15%)	0.70
CABG	19 (7%)	6 (8%)	5 (6%)	8 (8%)	0.86
*Dissection type:*					
Type A	123 (46%)	30 (38%)	46 (54%)	47 (47%)	0.12
Type B	144 (54%)	50 (63%)	40 (47%)	54 (54%)	
*Dissection management:*					
Surgery	116 (43%)	28 (35%)	43 (50%)	45 (45%)	0.14
Endovascular repair	40 (15%)	14 (18%)	10 (12%)	16 (16%)	0.54
Medical management	97 (36%)	34 (43%)	28 (33%)	35 (35%)	0.37
*Chronic medical therapy:*					
ARB	9 (7%)	2 (5%)	3 (8%)	4 (7%)	0.91
ACE-I	98 (38%)	28 (35%)	33 (40%)	37 (37%)	0.85
Beta-blocker	254 (96%)	76 (96%)	83 (97%)	95 (96%)	0.98
Calcium channel blocker	131 (50%)	39 (49%)	45 (54%)	47 (48%)	0.71
Statin	21 (26%)	3 (14%)	9 (41%)	9 (24%)	0.14
Diuretic	64 (46%)	18 (43%)	18 (49%)	28 (46%)	0.88

Abbreviations: ACE-I, angiotensin-converting enzyme inhibitor; ARB, angiotensin-receptor blocker; CABG, coronary artery bypass graft; PCI, percutaneous coronary intervention.

**Table 2:** Patient characteristics stratified by proportion of interval follow-up clinic visits after acute aortic dissection

Variables	Overall ( *n* = 267)	< 33%, *n* = 75 (28%)	33–66%, *n* = 115 (43%)	> 66%, *n* = 77 (29%)	*p* -Value
*Demographics:*					
Age (y)	59.9 ± 14.3	61.4 ± 16.0	58.8 ± 13.6	60.2 ± 13.5	0.46
Female gender	91 (34%)	28 (37%)	38 (33%)	25 (33%)	0.78
Caucasian (vs. other)	202 (77%)	55 (74%)	82 (73%)	65 (87%)	0.07
Distance to home (miles)	70.9 ± 61.3	79.8 ± 71.5	71.0 ± 61.5	62.3 ± 48.9	0.22
*Past medical history:*					
Marfan syndrome	17 (6%)	0 (0%)	12 (10%)	5 (7%)	**0.02**
Hypertension	202 (76%)	58 (78%)	90 (79%)	54 (70%)	0.33
Aortic aneurysm	37 (14%)	11 (15%)	16 (14%)	10 (13%)	0.94
Coronary artery disease	81 (31%)	22 (31%)	40 (35%)	19 (25%)	0.34
Bicuspid aortic valve	10 (4%)	3 (4%)	5 (5%)	2 (3%)	0.79
Prior aortic dissection	21 (8%)	3 (4%)	12 (11%)	6 (8%)	0.30
Mitral valve disease	9 (6%)	1 (2%)	6 (10%)	2 (5%)	0.34
Diabetes	19 (7%)	7 (10%)	7 (6%)	5 (7%)	0.66
Current smoking	76 (36%)	26 (46%)	30 (33%)	20 (32%)	0.39
Cocaine abuse	13 (5%)	7 (10%)	4 (4%)	2 (3%)	0.11
Renal insufficiency	7 (5%)	2 (5%)	4 (7%)	1 (3%)	0.89
PCI	11 (5%)	6 (9%)	4 (4%)	1 (1%)	0.19
Aortic valve disease	29 (11%)	7 (10%)	14 (12%)	8 (11%)	0.84
Emphysema	24 (17%)	7 (16%)	10 (16%)	7 (19%)	0.92
CABG	19 (7%)	5 (7%)	9 (8%)	5 (7%)	0.93
*Dissection type:*					
Type A	123 (46%)	29 (39%)	60 (60%)	34 (34%)	0.17
Type B	144 (54%)	46 (61%)	58 (48%)	43 (56%)	
*Dissection management:*					
Surgery	116 (43%)	23 (31%)	56 (49%)	37 (48%)	**0.03**
Endovascular repair	40 (15%)	10 (13%)	17 (15%)	13 (17%)	0.83
Medical management	97 (36%)	37 (49%)	37 (32%)	23 (30%)	**0.02**
*Chronic medical therapy:*					
ARB	9 (7%)	1 (2%)	5 (8%)	3 (9%)	0.46
ACE-I	98 (38%)	26 (35%)	43 (38%)	29 (40%)	0.85
Beta-blocker	254 (96%)	70 (95%)	112 (97%)	72 (96%)	0.61
Calcium channel blocker	131 (50%)	35 (47%)	57 (50%)	39 (52%)	0.84
Statin	21 (26%)	6 (29%)	9 (23%)	6 (29%)	0.85
Diuretic	64 (46%)	18 (43%)	31 (51%)	15 (41%)	0.56

Abbreviations: ACE-I, angiotensin-converting enzyme inhibitor; ARB, angiotensin-receptor blocker; CABG, coronary artery bypass graft; PCI, percutaneous coronary intervention.

Patients were followed for a maximum of 5 years after initial discharge. Mean follow-up was 54.7 ± 13.3 months overall, and there were 52 deaths. In the subgroup with Type A AAD ( *n* = 123), mean follow-up was 57.7 ± 7.9 months with 13 deaths, while in the subgroup with Type B AAD ( *n* = 144), mean follow-up was 52.2 ± 16.1 months with 39 deaths.

**Fig. 1:**
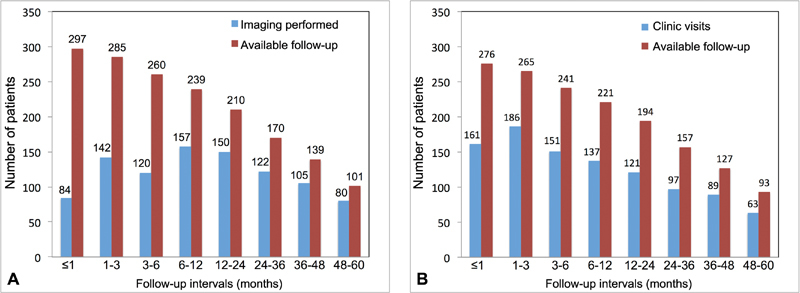
Frequency of aortic imaging follow-up ( **A** ) and clinic visits ( **B** ) at each recommended interval. The bars represent the number of patients with clinic visit and imaging follow-up for each interval, among the patients with available follow-up. As patients are censored from the study, the number of patients with available follow-up decreases.

**Fig. 2:**
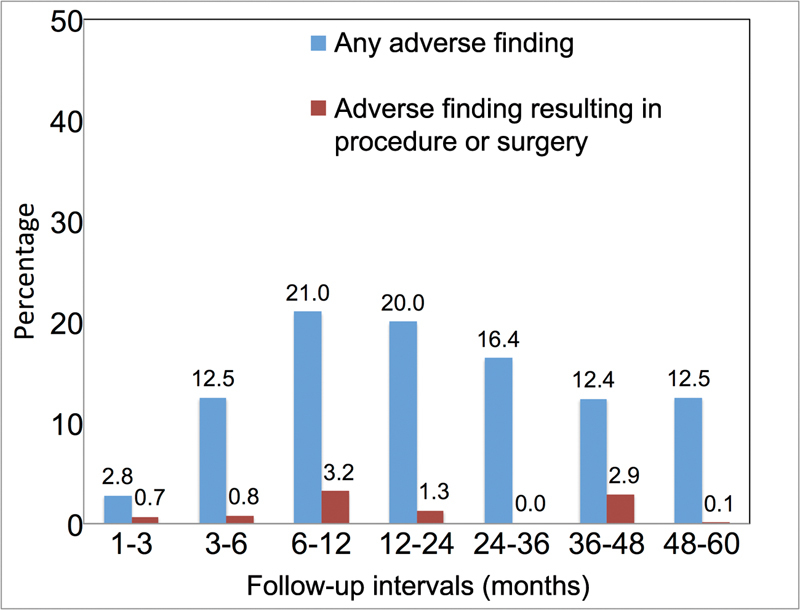
Frequency of adverse findings on imaging at each interval. The bars represent the number of imaging studies with adverse findings for each interval and the number of adverse imaging findings that are documented to prompt interventional procedures.

Figure 1 demonstrates the observed frequency of imaging surveillance and clinic visit follow-up for each time interval. CT was utilized in 94.5% of cases, magnetic resonance imaging (MRI) in 4.6% of cases, and TEE alone in 1.0% of cases. Table 3 provides the incidence of adverse imaging findings among the proportion of patients with imaging performed at each recommended interval, peaking between 6 and 12 months. Of these adverse findings, CT identified all but three cases, with the remainder reported by MRI. There were a total of 13 interventional procedures or surgeries prompted by adverse imaging findings during the period of follow-up ( Table 4 ). Rates of adverse imaging findings that resulted in a procedure or surgery were low, with the highest rate observed between 6 and 12 months after discharge ( Fig. 2 ). Tables 5 and 6 provide the number of imaging studies and clinic visits for patients in each of the three groups.

**Table 3:** Frequency of new adverse imaging findings at each interval

	Imaging interval (mo)						
Adverse imaging findings	> 1–3, *n* = 134	> 3–6, *n* = 111	> 6–12, *n* = 147	> 12–24, *n* = 139	> 24–36, *n* = 111	> 36–48, *n* = 98	> 48–60, *n* = 74
Increased false lumen size	−	−	4	7	3	3	1
New partial thrombosis of false lumen	1	4	6	2	1	1	−
Aortic dilatation or new aneurysm	1	9	25	20	16	7	8
New endograft leak	−	−	2	−	−	−	−
New dissection	−	−	1	−	−	−	1
Intramural hematoma	−	−	1	−	−	−	−
Pseudoaneurysm	1	−	−	−	−	1	−
Penetrating ulcer expansion	−	2	−	−	−	−	−
New dissection expansion into branch vessel	−	−	−	−	−	−	−
Other cardiovascular finding	1	−	−	1	1	−	1

Note: The total number of adverse findings is provided in Fig. 2.

**Table 4:** Procedures performed following adverse imaging findings

Case number	Initial dissection type	Adverse imaging findings	Treatment
1	B	New Type A AAD	Surgical replacement
2	A	Enlarging arch and descending aorta	Surgical replacement
3	A	Enlarging descending aorta	Endovascular repair
4	A	Enlarging proximal descending aorta	Surgical replacement
5	A	New occlusion of common carotid from false lumen	Surgical bypass
6	B	Enlarging arch	Surgical replacement
7	B	Enlarging arch and descending aorta	Surgical replacement
8	B	Enlarging descending aorta	Surgical replacement
9	B	Enlarging descending aorta	Surgical replacement
10	B	Enlarging ascending aorta and arch	Surgical replacement
11	B	Enlarging abdominal aneurysm	Surgical replacement
12	B	Enlarging descending aorta	Surgical replacement
13	B	Enlarging descending aorta	Surgical replacement

Abbreviation: AAD, acute aortic dissection.

Note: Since the patients were enrolled between 1996 and 2011, treatment approaches may not be consistent with contemporary practice.

**Table 5:** Surveillance imaging at each follow-up time interval

Follow-up time interval (mo)	< 33%, *n* = 80 (30%)	33–66%, *n* = 86 (32%)	> 66%, *n* = 101 (38%)	*p* -Value
≤1	19 (23.8%)	30 (24.8%)	23 (35.9%)	0.192
> 1–3	29 (36.3%)	69 (57.0%)	36 (57.1%)	0.008
> 3–6	22 (27.5%)	49 (41.2%)	40 (64.5%)	< 0.001
> 6–12	20 (25.0%)	72 (61%)	55 (91.7%)	< 0.001
> 12–24	9 (12.3%)	77 (67.5%)	53 (94.6%)	< 0.001
> 24–36	6 (9.1%)	54 (50.0%)	51 (94.4%)	< 0.001
> 36–48	3 (5.4%)	51 (52.0%)	44 (97.8%)	< 0.001
> 48–60	2 (4.1%)	36 (38.7%)	36 (92.3%)	< 0.001

**Table 6:** Follow-up clinic visits at each time interval

Follow-up time interval (mo)	< 33%, *n* = 75 (28%)	33–66%, *n* = 155 (43%)	> 66%, *n* = 77 (29%)	*p* -Value
≤1	30 (43.5%)	67 (55.4%)	56 (74.7%)	0.001
> 1–3	38 (55.1%)	83 (68.6%)	60 (81.1%)	0.004
> 3–6	16 (23.2)	75 (62.5)	53 (73.6)	< 0.001
> 6–12	9 (13.0%)	63 (53.4%)	58 (81.7%)	< 0.001
> 12–24	3 (4.7%)	55 (49.5%)	61 (89.7%)	< 0.001
> 24–36	1 (1.8%)	30 (28.0%)	60 (92.3%)	< 0.001
> 36–48	0	33 (33.7%)	46 (90.2%)	< 0.001
> 48–60	1 (2.4%)	25 (27.2%)	34 (72.3%)	< 0.001

In the subgroup of patients with imaging performed in at least 4 of the 8 recommended intervals ( *n* = 126), adverse imaging findings were observed in 2.8% (2/72) at 1 to 3 months, 14.0% (9/64) at 3 to 6 months, 27.0% (27/100) at 6 to 12 months, 18.4% (19/103) at 12 to 24 months, 17.0% (16/94) at 24 to 36 months, 9.8% (9/92) at 36 to 48 months, and 14.1% (10/71) at 48 to 60 months.

**Fig. 3:**
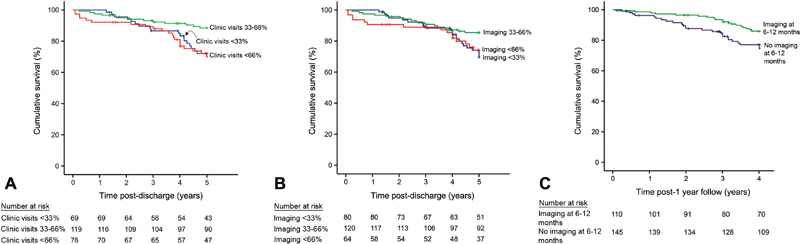
( **A** – **C** ) Kaplan–Meier curves for all-cause mortality in patients with acute aortic dissection stratified by frequency of imaging ( **A** ), clinic visit follow-up ( **B** ), and imaging between 6 and 12 months ( **C** ). The number at risk is provided below each image. For *p* -values, please refer the “Results” section.

In patients stratified by < 33%, 33 to 66%, or > 66% completion of interval imaging surveillance tests ( Fig. 3A ) and follow-up clinic visits ( Fig. 3B ), significant differences in unadjusted all-cause mortality were observed between groups for completion of recommended follow-up clinic visits ( *p* = 0.007) but not for completion of recommended imaging surveillance tests ( *p* = 0.10).

Compared with those with < 33% of imaging tests, multivariate analysis observed that patients with more frequent imaging did not have a difference in survival ( Table 7 ). In comparison to those with < 33% of clinic visits, patients with 33 to 66% of clinic visits had lower mortality on multivariable analysis, although no difference was seen in those with > 66% of clinic visits ( Table 8 ).

**Table 7:** Adjusted all-cause mortality in patients with acute aortic dissection stratified by frequency of surveillance imaging

Imaging frequency	HR	95% CI	*p* -Value
Imaging < 33%	1.0 (baseline)		−
Imaging 33–66%	0.66	0.34–1.28	0.22
Imaging > 66%	1.11	0.56–2.17	0.77

Abbreviations: CI, confidence interval; HR, hazards radio.

Note: *c* -Statistic = 0.67.

**Table 8:** Adjusted all-cause mortality in patients with acute aortic dissection stratified by frequency of follow-up clinic visits

Clinic visit frequency	HR	95% CI	*p* -Value
Clinic visit < 33%	1.0 (baseline)		−
Clinic visit 33–66%	0.47	0.23–0.97	0.04
Clinic visit > 66%	1.26	0.67–2.38	0.47

Abbreviations: CI, confidence interval; HR, hazards radio.

Note: *c* -Statistic = 0.73.

Patients with any imaging between 6 and 12 months (vs. none) had lower mortality ( *p* < 0.001) on unadjusted analysis ( Fig. 3C ), while imaging at other time intervals was not associated with a difference in mortality ( *p* > 0.05 for each). The presence versus absence of clinic visits at specific time intervals was not associated with any difference in mortality ( *p* > 0.05 for each). On multivariable analysis, imaging at 6 to 12 months was associated with improved survival (hazards ratio: 0.50, 95% confidence interval: 0.27–0.91, *p* = 0.02; *c* -statistic: 0.66). There were no significant differences in clinical characteristics between patients with and without adverse imaging findings, including the presence of Marfan syndrome, prior aortic aneurysm, prior aortic dissection, elevated systolic blood pressure, or elevated diastolic blood pressure ( *p* > 0.05 for each), which may be due to lack of statistical power.

Postdischarge blood pressure values were available for 93% (249/267) of patients. Between patients with < 33%, 33 to 66%, and > 66% clinic follow-up, there were no differences in mean systolic (131.9 ± 26.8 vs. 132.14 ± 17.3 vs. 127.9 ± 15.2 mm Hg, *p* = 0.30) or diastolic (71.6 ± 14.6 vs. 71.0 ± 11.2 vs. 71.6 ± 8.3 mm Hg, *p* = 0.92) blood pressure between groups. In patients with known baseline tobacco status ( *n* = 204), no difference in smoking between index hospitalization and follow-up was observed in those with < 33% clinic visit follow-up (43% [21/49] vs. 35% [17/49], *p* = 0.41), while a significant reduction in smoking was observed in those with 33 to 66% of clinic visits (37% [34/92] vs. 21% [19/92], *p* = 0.01) and > 66% of clinic visits (32% [20/63] vs. 16% [10/63], *p* = 0.04).

## Discussion

This study observes a wide range in the rate of surveillance imaging and clinic visit follow-up. Further, while adverse imaging findings were not uncommon, they rarely resulted in surgical or interventional procedures. Patients with intermediate rates of follow-up clinic visits after AAD had lower rates of mortality than patients with lower or higher rates of clinic visits. And finally, while the overall rate of surveillance imaging after AAD was not associated with mortality, imaging at 6 to 12 months was associated with improved survival. When coupled with our observation of peak adverse imaging findings and peak interventions for adverse imaging findings during this same interval, these results suggest that imaging between 6 and 12 months following discharge for AAD may represent an especially important period for surveillance imaging.

Existing guidelines endorse frequent follow-up, and recommend four clinic visits and surveillance imaging 3 4 studies in the first year, with annual follow-up thereafter in stable patients. Our results suggest that clinical practice is not entirely consistent with guidelines, although about a third of our cohort experienced AAD prior to their publication, 4 limiting our ability to determine adherence to guideline recommendations. The present results likely overestimate the actual rate of follow-up, as the study censored patients at the time of their last clinic visit or imaging follow-up for the respective analyses. This was done to reduce the possibility of underestimating follow-up, as this study did not have permission to contact patients directly to determine which patients may have moved or transferred their postdissection care to other centers. Furthermore, it is likely that patterns of follow-up may be lower at outside centers specializing in aortic disease.

Frequent imaging is associated with increased costs, and in the case of CT may expose patients to potential risks such as ionizing radiation and contrast-induced nephropathy. These must be weighed against the likelihood of adverse imaging findings that alter management. This study observes that adverse imaging findings are not uncommon, with the bulk of these demonstrating an increase in aortic size, greater false lumen size, and new partial thrombosis of a false lumen. While these findings may convey prognostic significance, they rarely resulted in changes in patient management in this study, suggesting that the clinical benefit of frequent imaging may be small in a general cohort of individuals following AAD.

Our observation of a potential survival benefit related to imaging between 6 and 12 months after discharge makes sense given the increased rate of adverse imaging findings and associated interventions that we observed during this interval. These findings suggest that this may be an especially important period for surveillance imaging, although we may be underpowered to detect differences in mortality related to imaging during other intervals. In contrast, there was a lack of survival benefit based on the overall frequency of imaging surveillance. This suggests that we could consider a reduced frequency of overall imaging surveillance in some patients following AAD, with a possible targeted approach for imaging during potentially higher-risk intervals. Future research to identify populations and time intervals at higher risk is needed to determine the optimal frequency and timing of imaging surveillance. While we did not identify clinical characteristics that predicted adverse imaging findings, this may be due to a lack of statistical power.

While frequent clinic visit follow-up with physicians specializing in post-AAD management may not necessarily correlate with the small risks inherent in imaging, it may also be associated with increased cost, and often requires significant patient travel due to the relatively small number of centers that specialize in post-AAD management. This study observed that patients with intermediate rates of clinic visits had the lowest adjusted mortality, suggesting a potential survival benefit related to regular follow-up in specialized clinics. Patients with higher rates of clinic visits had greater mortality, which could relate to unmeasured variables that may convey higher risk and prompt more frequent clinic follow-up. The higher mortality observed in patients with low rates of follow-up suggest a potential survival benefit of more frequent follow-up in specialized clinics, and suggest those with infrequent follow-up may be at higher risk. It is also possible that patients in the low follow-up group are noncompliant, and thus may be noncompliant with recommendations for other cardiac risk factors as well increasing the risk of mortality. Alternatively, patients with frequent follow-up may have higher risk features prompting more frequent follow-up. We observed no differences in blood pressure control between groups, which may represent adequate treatment by both the specialists and primary care providers for these individuals. While decreased tobacco smoking was observed in patients with increased clinic visit follow-up, this could also be due to more motivated patients who may have been more likely to return to clinic.

There are several limitations of this observational study. This was limited to review of the electronic medical record and the Social Security Death Index, and we could not directly contact patients to more completely establish follow-up. We acknowledge a potential selection bias due to exclusion of patients who may have been followed at other centers and undergone imaging elsewhere. To mitigate this limitation, we excluded patients who lived more than 500 miles away from our academic medical center, and censored patients at the time of the last clinic visit or imaging follow-up, which would be expected to overestimate follow-up. Further, there were univariate differences between groups stratified by rates of follow-up, and there may be unmeasured variables that we cannot account for. While we performed multivariable analysis to account for biases present on univariable analyses (such as differences in follow-up for patients with Marfan syndrome, prior cocaine use, and treated with medical management), we cannot exclude the possibility of incomplete multivariable adjustment and residual bias. Also, we limited follow-up to 5 years to have a more contemporary cohort, and we therefore cannot assess the effect of longer follow-up on events, and would miss delayed adverse events. Finally, we evaluated patients over a significant time interval to obtain an adequate sample size; there have been significant changes in practice patterns and recommendations during this time, which may be incompletely captured in our data.

Another limitation is that adverse imaging changes, such as false lumen enlargement or increased aortic dilatation, were based on any increase in size compared with the prior studies unless the radiologist felt this was within measurement error (in all cases within 1 mm from the prior study); while we considered the use of thresholds, the retrospective nature of this study with the use of clinical reports, and the lack of definitive thresholds, limit the utility of such an approach. We therefore decided to err on the side of including any potential adverse imaging findings and included any increase in size as an adverse event. Finally, this represents the experience of a single academic medical center. While this site is the largest enrollment site in the International Registry of Aortic Dissection, its experience may differ from other sites.

## Conclusions

We observed a wide range in the rate of specialized clinic visits and imaging surveillance following AAD that often did not match the frequency suggested in guidelines. While adverse imaging findings were not uncommon, they rarely resulted in management changes. We observed that patients with intermediate rates of clinic visits had lower mortality than those with lower or higher rates. Further, while the overall rate of surveillance imaging after AAD did not appear to impact mortality, imaging at 6 to 12 months was associated with improved mortality, which corresponds to peak rates in adverse imaging findings and associated interventions. These findings may improve our ability to optimize the frequency and timing of specialized clinic visits and surveillance imaging following AAD. Future prospective research comparing guideline-based follow-up to less frequent follow-up could be considered in patients at lower risk of complications.
